# COVID-19 biomarkers for severity mapped to polycystic ovary syndrome

**DOI:** 10.1186/s12967-020-02669-2

**Published:** 2020-12-22

**Authors:** Abu Saleh Md Moin, Thozhukat Sathyapalan, Stephen L. Atkin, Alexandra E. Butler

**Affiliations:** 1 Diabetes Research Center (DRC), Qatar Biomedical Research Institute (QBRI), Hamad Bin Khalifa University (HBKU), Qatar Foundation (QF), PO Box 34110, Doha, Qatar; 2 Academic Endocrinology, Diabetes and Metabolism, Hull York Medical School, Hull, UK; 3 Royal College of Surgeons in Ireland Bahrain, Adliya, Kingdom of Bahrain

To the Editor,

Large scale multi-omics analysis has identified significant differences in the biomarkers between COVID-19 disease and control subjects [1]. These protein panels target biological processes involved in vessel damage, platelet degranulation, the coagulation cascade and the acute phase response [1], with greater protein changes dependent on the COVID-19 severity. However, it is observed that in metabolic conditions such as polycystic ovary syndrome expressed proteins differ compared to control women [2] and PCOS patients have increased platelet aggregation and decreased plasma fibrinolytic activity, resulting in a prothrombotic propensity [3, 4], with elevated coagulation markers [5]. Therefore, any biomarkers reflecting COVID-19 disease and its severity would necessarily have to be independent of differentially-expressed proteins relating to other conditions; therefore, this proteomic analysis was undertaken in women with and without PCOS to compare with the proteomic biomarkers recently described in COVID-19 using shotgun proteomics followed by parallel reaction monitoring [1].

146 PCOS and 97 control women who presented sequentially to the Department of Endocrinology, Hull and East Yorkshire Hospitals NHS Trust were recruited to the local PCOS biobank (ISRCTN70196169) [2]. PCOS diagnosis was based on all three Rotterdam consensus diagnostic criteria. Proteins that were identified as being altered in COVID-19 disease for vessel damage (16 proteins), platelet degranulation (11 proteins), coagulation cascade (24 proteins) and acute phase response (9 proteins), shown in Table 1, were determined by Slow Off-rate Modified Aptamer (SOMA)-scan plasma protein measurement [6]. Statistics were performed using Graphpad Prism 8.0.

**Table 1:** Proteins identified as being altered in COVID-19 disease categorized according to biological processes: vessel damage (16 proteins), platelet degranulation (11 proteins), coagulation cascade (24 proteins) and acute phase response (9 proteins) in PCOS and control women

Target full name	Target	UniProt	Entrez gene ID	Entrez gene symbol	T-test PCOS vs control
Vessel damage					
Angiotensinogen	Angiotensinogen	P01019	183	AGT	0.0240
Angiopoietin-1	Angiopoietin-1	Q15389	284	ANGPT1	0.0760
Angiogenin	Angiogenin	P03950	283	ANG	0.2540
EGF-containing fibulin-like extracellular matrix protein 1	FBLN3	Q12805	2202	EFEMP1	0.4220
Gelsolin	Gelsolin	P06396	2934	GSN	0.0002
Hemopexin	Hemopexin	P02790	3263	HPX	0.4390
Inter-alpha-trypsin inhibitor heavy chain H4	ITI heavy chain H4	Q14624	3700	ITIH4	0.5360
Lumican	Lumican	P51884	4060	LUM	0.3790
Nidogen-1	Nidogen	P14543	4811	NID1	0.9600
Neuropilin-1	NRP1	O14786	8829	NRP1	0.0090
Periostin	Periostin	Q15063	10631	POSTN	0.0490
Ras-related C3 botulinum toxin substrate 1	RAC1	P63000	5879	RAC1	0.5850
Kallistatin	Kallistatin	P29622	5267	SERPINA4	0.0006
Pigment epithelium-derived factor	PEDF	P36955	5176	SERPINF1	0.00003
Transforming growth factor-beta-induced protein ig-h3	BGH3	Q15582	7045	TGFBI	0.9890
Tenascin	Tenascin	P24821	3371	TNC	0.0020
Vitronectin	Vitronectin	P04004	7448	VTN	0.3930
Platelet degranulation					
Alpha-2-macroglobulin	a2-Macroglobulin	P01023	2	A2M	0.2660
Clusterin	Clusterin	P10909	1191	CLU	0.6900
Fibronectin	Fibronectin	P02751	2335	FN1	0.0098
Platelet glycoprotein Ib alpha chain	GP1BA	P07359	2811	GP1BA	0.1560
Histidine-rich glycoprotein	HRG	P04196	3273	HRG	0.5890
Integrin alpha-IIb: beta-3 complex	gpIIbIIIa	P08514 P05106	3674 3690	ITGA2B ITGB3	0.8370
Neutrophil-activating peptide 2	NAP-2	P02775	5473	PPBP	0.2740
Plasma serine protease inhibitor	PCI	P05154	5104	SERPINA5	0.0380
Corticosteroid-binding globulin	CBG	P08185	866	SERPINA6	0.1790
Thyroxine-binding globulin	Thyroxine-Binding Globulin	P05543	6906	SERPINA7	0.5730
Transgelin-2	Transgelin-2	P37802	8407	TAGLN2	0.8690
von Willebrand factor	vWF	P04275	7450	VWF	0.0770
Coagulation cascade					
Carboxypeptidase B2	TAFI	Q96IY4	1361	CPB2	0.0170
Prothrombin	Prothrombin	P00734	2147	F2	0.0920
Coagulation Factor V	Coagulation Factor V	P12259	2153	F5	0.3020
Coagulation factor VII	Coagulation Factor VII	P08709	2155	F7	0.2520
Coagulation factor IX	Coagulation Factor IX	P00740	2158	F9	< 0.00001
Coagulation factor Xa	Coagulation Factor Xa	P00742	2159	F10	0.1620
Coagulation Factor XI	Coagulation Factor XI	P03951	2160	F11	0.5190
Fibrinogen	Fibrinogen	P02671 P02675 P02679	2243 2244 2266	FGA FGB FGG	0.0126
D-dimer	D-dimer	P02671 P02675 P02679	2243 2244 2266	FGA FGB FGG	0.00002
Fibrinogen gamma chain	Fibrinogen g-chain dimer	P02679	2266	FGG	0.00002
Hepatocyte growth factor activator	HGFA	Q04756	3083	HGFAC	0.0830
Plasma kallikrein	Prekallikrein	P03952	3818	KLKB1	0.0015
Kininogen-1	Kininogen, HMW	P01042	3827	KNG1	0.7970
Plasminogen	Plasminogen	P00747	5340	PLG	0.6740
Vitamin K-dependent protein S	Protein S	P07225	5627	PROS1	0.00003
Vitamin K-dependent protein C	Protein C	P04070	5624	PROC	0.5110
Alpha-1-antitrypsin	a1-Antitrypsin	P01009	5265	SERPINA1	0.1500
Protein Z-dependent protease inhibitor	protein Z inhibitor	Q9UK55	51156	SERPINA10	0.1720
Antithrombin-III	Antithrombin III	P01008	462	SERPINC1	0.0003
Heparin cofactor 2	Heparin cofactor II	P05546	3053	SERPIND1	0.00003
Plasminogen activator inhibitor 1	PAI-1	P05121	5054	SERPINE1	0.1680
Alpha-2-antiplasmin	a2-Antiplasmin	P08697	5345	SERPINF2	
Acute phase response					
Serum albumin	Albumin	P02768	213	ALB	0.0660
Macrophage mannose receptor 1	Macrophage mannose receptor	P22897	4360	MRC1	0.0020
Hepatocyte growth factor-like protein	MSP	P26927	4485	MST1	0.7340
Protein S100-A9	calgranulin B	P06702	6280	S100A9	0.0156
Serum amyloid A-1 protein	SAA	P0DJI8	6288	SAA1	0.5095
Alpha-1-antichymotrypsin	a1-Antichymotrypsin	P01011	12	SERPINA3	0.2672
Superoxide dismutase [Cu–Zn]	SOD	P00441	6647	SOD1	0.9448
Serotransferrin	Transferrin	P02787	7018	TF	0.0185
Transketolase	Transketolase	P29401	7086	TKT	0.8309

As reported previously [2], cohorts were age-matched, but PCOS women had increased insulin resistance, androgens and CRP (p < 0.001): systolic and diastolic blood pressure, and waist circumference were higher (p < 0.05).

For the 60 protein biomarkers described previously, 21 were found to differ in PCOS: for vessel damage 7 of 16 proteins differed, for platelet degranulation 2 of 11 proteins differed, for the coagulation cascade 9 of 24 proteins differed and for the acute phase response 3 of 9 proteins differed (Table 1).

These data show that altered protein expression relative to controls may occur in other conditions such as PCOS, and that COVID-19 biomarker changes found between respiratory patients with and without COVID-19 require validation before they can be confirmed to be related to COVID-19 disease and its severity. It is of concern that a number of significant protein biomarkers described for COVID-19 patients and its severity were also found in PCOS, spanning the biological processes involved in vessel damage, platelet degranulation, the coagulation cascade and the acute phase response, perhaps indicating why these patients may be at risk of more severe COVID-19 disease [7].

Limitations of the study include utilizing a different method of proteomic analysis compared to others that may not be directly comparable [1].

In conclusion, 21 of 60 protein biomarkers reported in respiratory patients with COVID-19 were found to differ between women with and without PCOS, showing the necessity for validation of such biomarkers, and suggesting that more severe COVID-19 disease may occur in PCOS.

## Data Availability

All the data for this study will be made available upon reasonable request to the corresponding author.
