# Correction to: COVID-19 biomarkers for severity mapped to polycystic ovary syndrome

**DOI:** 10.1186/s12967-021-02782-w

**Published:** 2021-03-15

**Authors:** Abu Saleh Md Moin, Thozhukat Sathyapalan, Stephen L. Atkin, Alexandra E. Butler

**Affiliations:** 1 Diabetes Research Center (DRC), Qatar Biomedical Research Institute (QBRI), Hamad Bin Khalifa University (HBKU), Qatar Foundation (QF), PO Box 34110, Doha, Qatar; 2 Academic Endocrinology, Diabetes and Metabolism, Hull York Medical School, Hull, UK; 3 Royal College of Surgeons in Ireland Bahrain, Adliya, Kingdom of Bahrain

## Correction to: J Transl Med (2020) 18:490 https://doi.org/10.1186/s12967-020-02669-2

Following publication of the original article [1], the authors would like to correct the author group with regards to the equal contributions: Stephen L. Atkin and Alexandra E. Butler should be listed as joint senior authors.

The author group has been updated above and the original article [1] has been corrected.
